# A Comparison of the Behavior, Physiology, and Offspring Resilience of Gestating Sows When Raised in a Group Housing System and Individual Stalls

**DOI:** 10.3390/ani11072076

**Published:** 2021-07-12

**Authors:** Xin Liu, Pengkang Song, Hua Yan, Longchao Zhang, Ligang Wang, Fuping Zhao, Hongmei Gao, Xinhua Hou, Lijun Shi, Bugao Li, Lixian Wang

**Affiliations:** 1 Institute of Animal Science, Chinese Academy of Agricultural Sciences, Beijing 100193, China; firstliuxin@163.com (X.L.); pengkangsong2021@163.com (P.S.); zcyyh@126.com (H.Y.); zhlchias@163.com (L.Z.); ligwang@126.com (L.W.); zhaofuping@caas.cn (F.Z.); gaohongmei_123@126.com (H.G.); 7hxh73@163.com (X.H.); shilijun01@caas.cn (L.S.); 2 College of Animal Science, Shanxi Agricultural University, Jinzhong 030801, China

**Keywords:** group housing system, individual stall, behavior, stress hormone, offspring, gestating sows

## Abstract

**Simple Summary:**

The housing patterns of gestating sows affect their health and welfare. In this study, the differences between behavior and stress hormone levels were assessed when sows were housed in a group housing system compared to individual stalls; in addition, the disease resistance and resilience of their piglets were compared. In our investigation, the group-housed sows showed more exploratory behavior, less vacuum chewing, less sitting behavior, and lower stress hormone levels throughout pregnancy. A lipopolysaccharide (LPS) injection test revealed that the offspring of group-housed sows showed better resistance and resilience to disease. Therefore, the gestating sows raised in a group housing system and their piglets are healthier and have improved welfare. Our results show that a group housing system provides higher welfare standards, with conditions that are more suitable for gestating sows in modern pig production.

**Abstract:**

Being in a confined environment causes chronic stress in gestating sows, which is detrimental for sow health, welfare and, consequently, offspring physiology. This study assessed the health and welfare of gestating sows housed in a group housing system compared to individual gestation stalls. After pregnancy was confirmed, experimental sows were divided randomly into two groups: the group housing system (GS), with the electronic sow feeding (ESF) system; or individual stall (IS). The behavior of sows housed in the GS or IS was then compared; throughout pregnancy, GS sows displayed more exploratory behavior, less vacuum chewing, and less sitting behavior (*p* < 0.05). IS sows showed higher stress hormone levels than GS sows. In particular, at 41 days of gestation, the concentration of the adrenocorticotropic hormone (ACTH) and adrenaline (A) in IS sows was significantly higher than that of GS sows, and the A level of IS sows remained significantly higher at 71 days of gestation (*p* < 0.01). The lipopolysaccharide (LPS) test was carried out in the weaned piglets of the studied sows. Compared with the offspring of gestating sows housed in GS (PG) or IS (PS), PG experienced a shorter period of high temperature and showed a quicker return to the normal state (*p* < 0.05). Additionally, their lower levels of stress hormone (*p* < 0.01) suggest that PG did not suffer from as much stress as PS. These findings suggested that gestating sows housed in GS were more able to carry out their natural behaviors and, therefore, had lower levels of stress and improved welfare. In addition, PG also showed better disease resistance and resilience. These results will provide a research basis for the welfare and breeding of gestating sows.

## 1. Introduction

In most parts of the world, gestating sows face stress due to space and management during gestation in intensive pig production systems. Conventional individual stall housing (IS) is commonly used for gestating sows because it makes handling easier, has a low capital cost, and reduces social stress [1]. However, the space restrictions of stalls limit the innate behaviors of gestating sows; therefore, pigs housed in IS cannot execute the behaviors needed to meet their specific needs [2,3]. These housing deficiencies cause sows to exhibit abnormal behaviors and physiology, causing chronic disease and leading to a reduction in muscle strength and bone density [4,5]. In order to improve the welfare of gestating sows, this IS practice was banned by the European Union (CD 2001/88/EC), who instead promotes group housing systems (GS) in European countries. Sows housed in GS suffer less than those housed in IS. GS with an electronic sow feeding (ESF) system provided gestating sows with a less physiologically stressful environment and greater opportunities for activity [6]. However, GS also has some disadvantages; for example, individual feeding is more difficult, and sows can be more aggressive in the early stage of mixing, leading to more injuries [7,8]. In the Chinese pig industry, gestating sows are still reared in IS in almost all pig farms. With the modernization of the pig industry and the emphasis on animal welfare, GS may be the direction of development. Therefore, it is necessary to study the effects of different housing systems on sows to provide the pig industry with more information.

Previous studies have compared the effects of reproductive performance, management, and behaviors on gestating sows housed in different housing systems. Several studies showed that sow reproductive performance was improved in GS, with others confirming that no differences were found among housing types [8,9,10]. Some researchers recommended that gestating sows housed in GS showed an improved welfare status, greater levels of activity, and fewer physiological abnormalities, but some studies did not find a significant difference in stress-related hormones between the two housing conditions [6,10,11]. However, previous studies have reported conflicting results, and limited data have been garnered regarding piglet resilience. Therefore, it is necessary to compare the effects on the behavior, physiology, and piglet resilience of gestating sows when housed in GS or in IS.

The objective of this study was to assess the effects of GS and IS on the health and welfare of gestating sows and their offspring, by detecting sows’ behaviors, physiology, and offspring resilience. The hormonal and behavioral changes in gestating sows housed in GS or IS were observed throughout the gestation period, and the disease resistance and resilience of the piglets was detected using a lipopolysaccharide (LPS) injection model. The results of the study could provide the scientific support for improving the health and welfare of gestating sows and piglets in production.

## 2. Materials and Methods

### 2.1. Ethistall Statements

All methods and procedures in the study were carried out according to the standard guidelines on experimental animals (No. IASCAAS-AE-09), which were established by the Animal Ethical Committee of the Institute of Animal Science, Chinese Academy of Agricultural Sciences (IAS-CAAS) (Beijing, China). The experimental protocols were approved by the Science Research Department of IAS-CAAS (Beijing, China) (No. IAS2019-18).

### 2.2. Animals and Management

All experimental animals were Large White pigs reared in identical intensive breeding conditions (Chang Rong Nong Ke, Yuncheng, China), with the same feed and management. The nutrient requirements of sow and piglet diets refer to NRC 2012 (Nutrient Requirements of Swine of the National Research Council). A total of 60 experimental sows with the second parity were artificially inseminated; pregnancy was confirmed with an ultrasound analyzer within 28 days of insemination. Then, sows with a confirmed pregnancy were allotted to their housing groups‒30 sows in IS and 30 in the GS. They were all moved to the farrowing crate three days before the expected delivery.

The offspring piglets of the test sows were used for disease resistance and resilience tests. Twenty piglets, each born from sows housed in IS or GS, were used for disease resistance. Test piglets with good physical health (remaining healthy and free of illness) and similar weaning weights were weaned at 21 days of age.

### 2.3. Housing Systems

**Figure 1:**
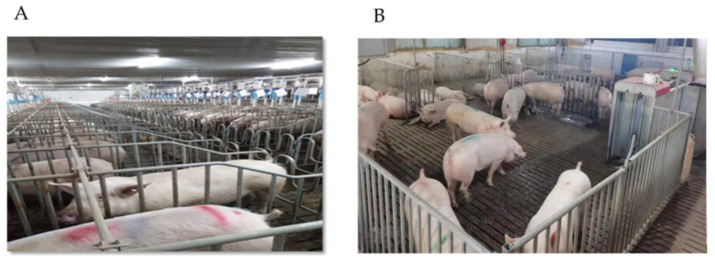
Types of housing facilities of sows. (**A**) Individual Stalls (IS). (**B**) Group system with ESF (GS).

In the study, the IS size was 2.40 m × 0.65 m (length × width, 1.56 m^2^/head) with an individual feeder and drinker (Figure 1A). The IS was slightly larger than the size of the sow’s body; there was only enough room for the sow to stand or lie down in place, with no room for the sow to turn around or move freely. The gestating sows of GS were housed in a room (10.5 m × 14.4 m, 5.04 m^2^/head) with an ESF, which provided enough space for the sows (Figure 1B). Sows in the group house could move freely, which allowed them to meet some of their innate behavioral requirements. The temperature of the gestating room was approximately 20 °C, which could be controlled using a fan or by heating.

### 2.4. Behavioral Observations

The behaviors of all experimental sows were recorded using a video surveillance system (Hikvision camera, Hangzhou, China) for data collection, which clearly recorded the movement of each experimental sow and avoided artificial observation errors. The gestating sows were continuously video recorded from 9:00 a.m. to 5:00 p.m. on each behavioral observation day (days 40, 70, and 100 of gestation). We observed and recorded the standing behavior, dog sitting behavior, lying down behavior, vacuum chewing behavior, and exploratory behavior of gestating sows; the definitions of various behaviors are listed in Table 1. The total number of instances of each behavior on the observation days was counted by recording the number of behaviors every ten minutes.

### 2.5. Sample Collection and Physiological Analysis

Blood samples were collected from the jugular vein of all experimental sows at days 41, 71, and 101 of gestation. The blood samples were kept at room temperature for 2 h and then the serum was separated and extracted by centrifugation at 3500 rpm for 10 min. The samples were stored at −80 °C. The samples were tested for the adrenocorticotropic hormone (ACTH), adrenaline (A) and cortisol (COR). ACTH and COR were measured through radioimmunoassay. Adrenaline was measured with the enzyme-linked immunosorbent assay (ELISA) method.

### 2.6. Disease Resilience Test of Piglets

Forty 21-day-old, 6 kg, healthy weaned piglets were selected for the experiment. Twenty of them were randomly selected from 10 litters of the test gestating sows housed in IS (PS), and the others were randomly selected from 10 litters of the test gestating sows housed in GS (PG). Two piglets were randomly selected from each litter and then assigned to lipopolysaccharide (LPS) and normal saline (NS) injection group, respectively. The injection dose of LPS (from *E. coli* O55:B5) or NS was 15 μg/kg BW. The ratio of male to female was half in each group. The ear temperature of piglets was measured with an animal thermometer at 1 h before injection, 1 h after injection, 2 h after injection, 3 h after injection, 4 h after injection, 5 h after injection, and 6 h after injection [14,15]. Blood was collected by jugular venipuncture 6 h after injection. Serum was extracted and frozen at −80 °C. The concentration of serum COR was measured. The determination method of COR was the same as 2.5.

### 2.7. Statistical Analysis

The collected data of behavioral and physiological tests were analyzed for the homogeneity of variance and then different significance analysis was carried out. These data were tested using *t*-test in SAS (version 9.2, SAS Inst. Inc., Raleigh, NC, USA). The results of the analysis were presented as the means ± standard error. The differences and statistical significance between groups were considered at *p* < 0.05 and *p* < 0.01.

## 3. Results

### 3.1. The Behavioral Response of Gestating Sows Housed in GS or IS

**Figure 2:**
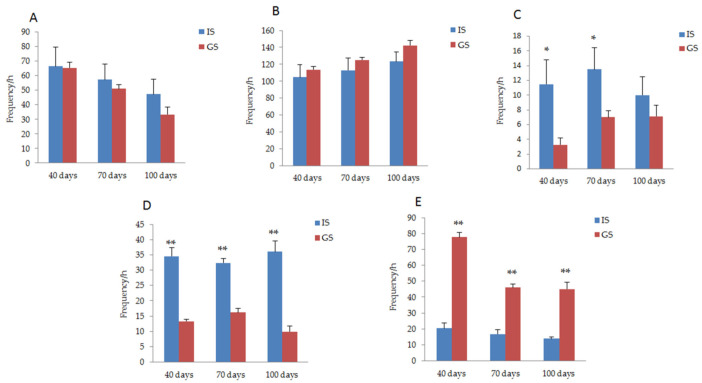
Comparison of the behavioral responses of gestating sows between IS and GS. (**A**) Changes in the standing behavior of gestating sows housed in IS and GS. (**B**) Changes in the lying down behavior of gestating sows housed in IS and GS. (**C**) Changes in the dog sitting behavior of gestating sows housed in IS and GS. (**D**) Changes in the vacuum chewing behavior of gestating sows housed in IS and GS. (**E**) Changes in the exploratory behavior of gestating sows housed in IS and GS. * *p* < 0.05. ** *p* < 0.01.

The behavioral response of gestating sows was compared between GS and IS groups, as shown in Figure 2. On days 40 and 70 of gestation, the frequency of dog sitting behavior in the gestating sows housed in IS was significantly higher than that in the GS condition (*p* < 0.05). During the whole pregnancy period, the frequency of empty chewing behavior in gestating sows housed in IS was significantly higher than that of sows in GS, while the frequency of exploratory behavior was significantly lower (*p* < 0.01). The frequency of standing behavior in gestating sows housed in the GS was less than that in sows housed in IS (*P*_40 day_ = 0.94, *P*_70 day_ = 0.58, *P*_100 day_ = 0.24), while the lying down behavior increased (*P*_40 day_ = 0.58, *P*_70 day_ = 0.43, *P*_100 day_ = 0.16); however, these behavioral differences did not reach a significant level.

### 3.2. Effects of IS or GS Housing Systems on the Physiological Responses of Gestating Sows

**Figure 3:**
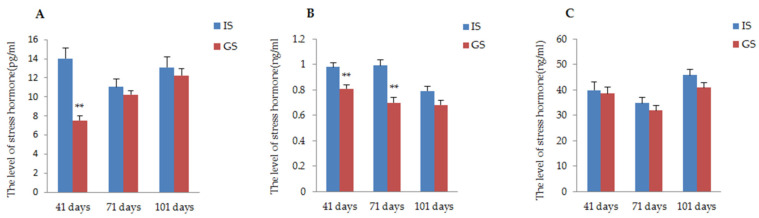
The influence of two different housing systems on the concentration of the physiological index in gestating sows. (**A**) Comparison of ACTH concentration of gestating sows raised in GS and IS; (**B**) Comparison of A concentration of gestating sows raised in GS and IS; (**C**) Comparison of COR concentration of gestating sows raised in GS and IS. ** *p* < 0.01.

The effects of the two different housing systems of gestating sows on physiological responses during gestation are presented in Figure 3. According to the data, the stress hormone (ACTH, A, COR) level of gestating sows housed in IS was higher than that of gestating sows housed in GS throughout the whole gestation period. Particularly, the concentrations of ACTH and A in gestating sows were significantly improved in IS compared to those reported in GS on day 41 of gestation; in addition, a significant increase in hormone A continued until day 71 of gestation (*p* < 0.01). The COR concentrations of sows in IS were numerically higher than the concentrations in GS sows, but this was not a significant difference (*P*_41 day_ = 0.75, *P*_71 day_ = 0.35, *P*_101 day_ = 0.09).

### 3.3. Comparison of Resistance and Resilience of Offspring Piglets

**Figure 4:**
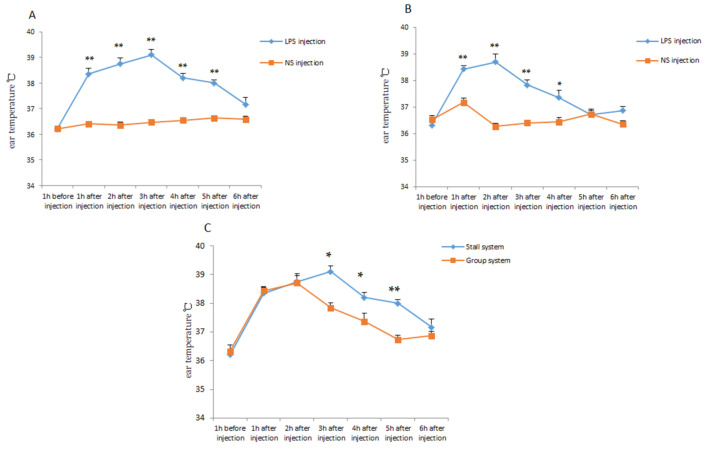
The ear temperature changes in the experiment of piglet resilience. (**A**), Changes of ear temperature of PS after NS or LPS injection. (**B**), Changes of ear temperature of PG after NS or LPS injection. (**C**), Changes of ear temperature of PG or PS after LPS injection. * *p* < 0.05. ** *p* < 0.01.

The model of inflammatory response was constructed by injecting LPS into piglets. The ear temperature of the piglets was measured before and after injection. As shown in Figure 4A,B, with NS injection as the control group, the ear temperature of the piglets was significantly higher after the LPS injection (*p* < 0.01). After the LPS injection, the ear temperature of the offspring piglet, both PG and PS, was raised rapidly and continued to return to normal 6 h after injection. It was also found that the duration of higher ear temperature of PS was longer than that of PG, in other words, the ear temperature of PG returned to normal state significantly faster and easier (*p* < 0.05) (Figure 4C). The concentration level of hormone COR of PG was significantly lower than that of PS (*p* < 0.01) (Table 2). These results indicated that the offspring piglets of gestating sows housed in the group system had greater resistance and resilience.

## 4. Discussion

In the present study, two different housing systems (individual stall and group housing) for gestating sows were compared. Gestating sows housed in IS had limited space, and GS sows had more freedom of movement. Floor space allowance markedly affects sow welfare [16], particularly during early gestation. Accordingly, appropriate housing is important to protect embryos and to confirm pregnancy [17]. The narrow space and metal-bars of the stall restrict the behaviors of gestating sows, particularly in late pregnant period of pregnancy, when the sow’s size and body weight increase [11]. IS housing is considered to be a chronic stressor for gestating sows, and has negative consequences on welfare and health [18]. Chronic stress has persistent effects on the behavior, physiology, and performance of sows and offspring [19,20]. The abnormal behavior of the sow not only reflects the response to environmental adaptability, but also the sow’s own psychological welfare. If sows are not comfortable during pregnancy, they will exhibit abnormal behaviors, for example, locomotion difficulties, stereotypies, etc., resulting in physiological and psychological stress [21]. In the present study, the postural behaviours of gestating sows in the two housing systems were compared. The frequency of standing in GS gestating sows was less than IS gestating sows, while the frequency of lying down was increased (though not at a significant level). GS sows exhibited more exploratory behavior and less vacuum chewing and dog sitting behavior. This suggested gestating sows housed in GS were healthier and had better welfare. Some previous studies showed similar results. Haley’s study [22] showed that sows were in a state of physical discomfort when they spent less time lying down and more time standing without eating. Confinement in stalls has been implicated in the development of oral stereotypies and repetitive, apparently functionless behaviors; the normal exploratory behavior of the sows could not be satisfied, mainly because of the extremely limited environmental stimuli [19,21]. Janssens’ study also demonstrated that sows in a group-housing system showed a decrease in the frequency of sham chewing and an increase in non-agonistic social behavior [23].

Stress impacts several physiological systems and the stress hormone levels have been used for physiological measurement [24]. Animal responses to stress activate the hypothalamic-pituitary-adrenocortical (HPA) axis and cause increased plasma levels of cortisol and catecholamines [25]. With an increased confinement duration, the sows in the restraining environment became bored and showed a failure response pattern by the activation of the HPA system [26]. Under chronic stress, the activation of the HPA system increased responsiveness of the adrenal cortex to ACTH and eventually lead to increased cortisol output [23]. In our study, the concentration of stress hormones (ACTH, A, and COR) in gestating sows housed in IS was higher than that in GS. Gestating sows housed in IS produced chronic long-term stress and increased stress hormone levels. Some studies have reported study similar results. Jang [6] reported that compared with the group sows, conventional stall sows had a higher serum cortisol level at 110 days of gestation. Merlot [27] showed that the conventional system was more stressful for sows during gestation, as illustrated by the elevated cortisol levels in the saliva of gestating sows; furthermore, the conventional system moderately worsened sow health in late gestation. In Quesnel’s study [28], sows raised in the conventional system had greater salivary concentrations of cortisol compared with the enriched system (larger pens and on deep straw bedding) during the gestation period. Optimizing commercial housing conditions would reduce stress levels and have positive effects on the immune status of mothers during gestation [29].

During the sow’s gestation period, the environment (including housing and management systems) can generate maternal stress, which can be detrimental to sow welfare and health, and also it could influence on off-spring physiology, such as the immune function, and impairing neonatal health [30,31,32]. Therefore, in order to continue the study of how maternal stress caused by different housing systems during the gestation period, affected their offspring, the piglet health and resilience test was designed and implemented. LPS is a major structural part of the outer membrane of Gram-negative bacteria and can effectively stimulate the body’s immune system. Therefore, LPS has been widely applied as an experimental model in vertebrate immune stress tests [33,34]. The acute phase response (APR) was induced by LPS stimulation and also caused the behavioral changes and physiological disorders in the pigs, including an elevated body temperature, increased cytokine levels, reduced feed intake, etc. [35]. In our test, we obtained the similar results. After LPS injection into piglets, APR was induced and their body temperature increased rapidly and significantly. Compared with PS in the test, PG experienced a shorter period of high temperature and the return to a normal state was faster; in addition, they suffered lower levels of stress in terms of their stress hormone levels. According to the concept of resilience, the ability of an animal to maintain performance under infection, or to rapidly return to prior performance levels after infection [36,37], PG was considered to have better resilience. Previous research reported stress piglets displayed higher levels of circulating cortisol [38], and with the same result, in the present study, PS suffered more stress in terms of their stress hormone levels. All of this suggested that the offspring of sows housed in GS during gestation had better resistance and resilience, which showed that these piglets were healthier. PS suffered with a higher level of stress and had lower resistance and resilience, which may be caused by the IS-housing-related stress experienced by their mothers during gestation.

## 5. Conclusions

Gestating sows were exposed to different environments and faced different challenges when they were housed in two systems (IS and GS). As a result of enjoying a more relaxed and comfortable environment, the sows housed in GS wereconductive more as in accordance with to their nature. Gestating sows housed in GS demonstrated more exploratory behavior, less vacuum chewing, and less dog sitting behavior compared with IS sows. Meanwhile, GS sows had a lower concentration of stress hormone than IS. In addition, the results of LPS injection experiment showed that PG had better resistance and resilience than PS. These findings provide a research basis for welfare breeding of gestating sows.

## Figures and Tables

**Table 1:** Behavior categories of pregnant sows and their definitions.

Behavior Categories	Definitions
Standing behavior	All four hooves are on the pen floor with limbs extended or the pig is walking with limbs in both extension and flexion and moving throughout the pen [12]
Dog sitting behavior	The front limbs are extended and bearing weight the rear limbs and body are in contact with the pen floor [12]
Lying down behavior	The pig’s body and limbs are in contact with the pen floor [12]
Vacuum chewing behavior	Continuous chewing while no feed is present in the mouth [8]
Exploratory behavior	Actively manipulating and exploring the surrounding environment [13]

**Table 2:** The concentration of COR in piglets in LPS injection test.

Piglet	Hormone	NS Injection	LPS Injection	*p*-Value
PS	COR (ng/mL)	72.30 ± 14.27 ^A^	158.34 ± 13.50 ^a,C^	0.0003
PG	COR (ng/mL)	32.79 ± 10.77 ^B^	112.74 ± 21.08 ^b,c^	0.0003

^A^,^a^,^B^,^b^: Means in the same row with different superscripts are significantly different (*p* < 0.01). ^C^,^c^: Means in the same column with different superscripts are significantly different (*p* < 0.01).
